# Berberine Inhibits HIV Protease Inhibitor-Induced Inflammatory Response by Modulating ER Stress Signaling Pathways in Murine Macrophages

**DOI:** 10.1371/journal.pone.0009069

**Published:** 2010-02-09

**Authors:** Weibin Zha, Guang Liang, Jian Xiao, Elaine J. Studer, Phillip B. Hylemon, William M. Pandak,, Guangji Wang, Xiaokun Li, Huiping Zhou

**Affiliations:** 1 Department of Microbiology & Immunology and Internal Medicine/Gastroenterology and McGuire Veterans Affairs Medical Center, Virginia Commonwealth University, Richmond, Virginia, United States of America; 2 China Pharmaceutical University, Nanjing, China; 3 School of Pharmacy, Wenzhou Medical College, Wenzhou, China; University of Maryland School of Pharmacy, United States of America

## Abstract

**Background:**

HIV protease inhibitor (PI)-induced inflammatory response plays an important role in HIV PI-associated dyslipidemia and cardiovascular complications. This study examined the effect of berberine, a traditional herb medicine, on HIV PI-induced inflammatory response and further investigated the underlying cellular/molecular mechanisms in macrophages.

**Methodology and Principal Findings:**

Cultured mouse J774A.1 macrophages and primary mouse macrophages were used in this study. The expression of TNF-α and IL-6 were detected by real-time RT-PCR and ELISA. Activations of ER stress and ERK signaling pathways were determined by Western blot analysis. Immunofluorescent staining was used to determine the intracellular localization of RNA binding protein HuR. RNA-pull down assay was used to determine the association of HuR with endogenous TNF-α and IL-6. Berberine significantly inhibited HIV PI-induced TNF-α and IL-6 expression by modulating ER stress signaling pathways and subsequent ERK activation, in turn preventing the accumulation of the RNA binding protein HuR in cytosol and inhibiting the binding of HuR to the 3′-UTRs of TNF-α and IL-6 in macrophages.

**Conclusions and Significance:**

Inhibition of ER stress represents a key mechanism by which berberine prevents HIV PI-induced inflammatory response. Our findings provide a new insight into the molecular mechanisms of berberine and show the potential application of berberine as a complimentary therapeutic agent for HIV infection.

## Introduction

Human immunodeficiency virus (HIV) protease inhibitors (PIs) have been used successfully in highly active anti-retroviral therapy (HAART) for HIV infection. Incorporation of HIV PIs in HAART causes profound and sustained suppression of viral replication, significantly reduces the morbidity and mortality, and prolongs the lifespan of patients with HIV infection [1]. However, accumulating clinical evidence indicates that HAART has changed the clinical profile of HIV infection from a sub-acute lethal disease to a chronic ambulatory disease [2]. Dyslipidemia specifically associated with HIV PIs has become a matter of particular concern in the clinic [3], [4]. More than 50% of patients receiving HAART develop dyslipidemia, which is a well-known risk factor for serious cardiovascular complications including endothelial dysfunction and atherosclerosis [5]. Although the mechanism underlying HIV PI-induced atherosclerosis remains to be fully identified, an increasing body of evidence suggests that multiple mechanisms may be involved, and individual PIs may have different effects on lipid metabolism. The development of other therapeutic interventions to effectively counteract the HIV PI-associated complications is especially urgent.

Numerous pieces of evidence from both animal models and human specimens suggest that inflammation represents one of the major events in the pathophysiology of atherosclerosis [6]. In addition to the accumulation of free cholesterol in macrophages, another key characteristic of atherosclerotic lesions is the presence of abundant inflammatory cytokines [7]. We have previously demonstrated that HIV PIs induce the accumulation of intracellular free cholesterol, deplete the endoplasmic reticulum (ER) calcium (Ca^2+^) stores, induce the ER stress and subsequent activation of the unfolded protein response (UPR), and promote foam cell formation in macrophages [8]. Our most recent studies have shown that HIV PIs promote the inflammatory response by regulating RNA binding protein HuR *via* the ERK signaling pathway in macrophages [9], [10]. Other investigators have suggested that HIV PI-induced ER stress response represents an important cell signaling mechanism underlying HIV PI-induced metabolic syndromes [11]. These observations indicate that, similar to lipid-lowering drugs, anti-inflammatory agents and ER-stress modulators may be useful for the treatment of HIV PI-induced cardiovascular complications and metabolic syndromes.

Berberine, an isoquinoline alkaloid isolated from many medicinal herbs, such as the Chinese herb *Huanglian*, *berberis aquifolium*, and *berberis vulgaris*, has been employed in traditional Chinese medicine to treat various infectious disorders for more than 3,000 years [12]. During the last few decades, many studies have shown that berberine has various beneficial effects on the cardiovascular system and significant anti-inflammatory activities [13]. It also has been identified as a novel cholesterol-lowering drug through a unique mechanism distinct from the current statin therapy [14]. Furthermore, the most recent study demonstrated that berberine suppresses the pro-inflammatory response and improves obesity-induced lipid dysregulation by modulating AMP-dependent protein kinase (AMPK) activity [15], [16].

An increasing amount of attention has been paid to the use of complementary and alternative medicine as a part of the treatment for HIV infection and the complications associated with HAART [17]. However, the scientific evidence for the use of alternative medicines is still lacking and the molecular targets of these beneficial effects of natural products have not been revealed. In the present study, we examined the potential protective effect of berberine on HIV PI-induced inflammatory response in macrophages. The results presented herein indicate that inhibition of ER stress represents a key mechanism by which berberine prevents HIV PI-induced inflammatory response. Our findings provide a new insight into the molecular mechanisms of berberine and show the potential application of berberine as a complimentary therapeutic agent for HIV infection.

## Results

### Effect of Berberine on HIV PI-Induced TNF-α and IL-6 Expression in Macrophages

**Figure 1:**
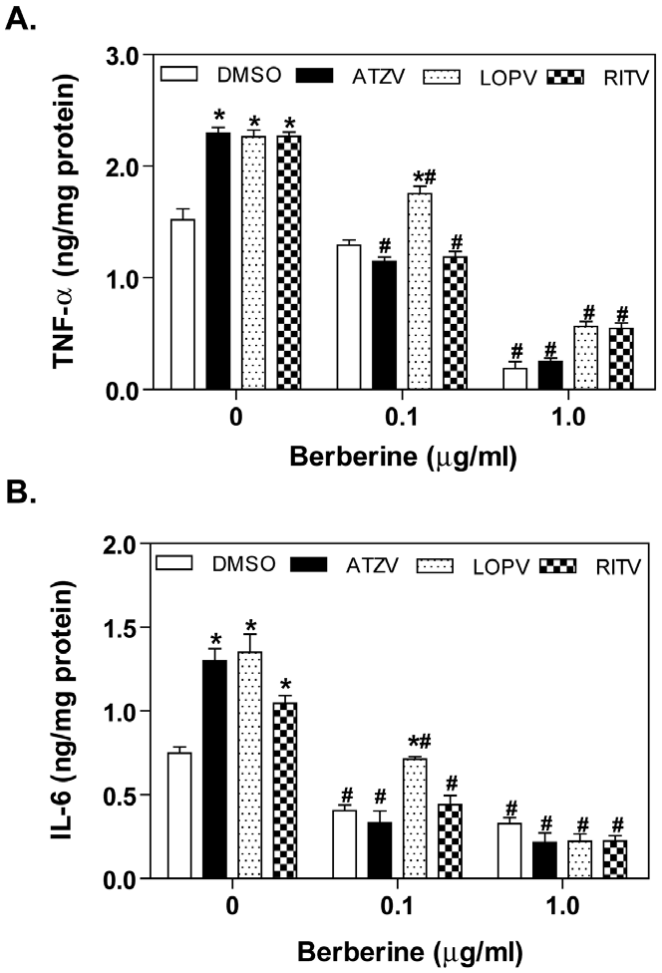
Effect of berberine on HIV PI-induced TNF-α and IL-6 expression in J774A.1 macrophages. Mouse J774A.1 cells were pretreated with berberine (BBR, 0.1 or 1.0 µg/ml) for 2 h, then treated with DMSO, atazanavir (ATZV, 15 µM), lopinavir (LOPV, 15 µM), or ritonavir (RITV, 15 µM) for 24 h. At the end of treatment, cell culture medium was collected. The protein levels of TNF-α and IL-6 were determined by ELISA as described in “Methods”. Values are mean ± SD of 3 independent experiments. *p< 0.05, statistical significance relative to vehicle control, DMSO. ^#^ *p*<0.05, statistical significance of the same HIV PI treatment between control group and BBR group. A. TNF-α; B. IL-6.

Accumulation of inflammatory cytokines is a major characteristic of atherosclerotic lesions. It has been well-demonstrated that TNF-α and IL-6 are mostly secreted by macrophages [7]. Our previous studies demonstrated that HIV PIs, atazanavir, lopinavir and ritonavir markedly increased TNF-α and IL-6 expression in macrophages, but amprenavir had no effect [9]. Numerous studies have reported that berberine has anti-inflammatory effects both *in vitro* and *in vivo* [13] and the most recent studies also have shown that berberine effectively inhibited lipopolysaccharide (LPS)-induced TNF-α and IL-6 expression [16]. First, we examined whether berberine is able to inhibit HIV PI-induced TNF-α and IL-6 expression in macrophages. Mouse J774A.1 cells were pre-treated with berberine (0.1 or 1.0 µg/ml) for 2 h, and then treated with vehicle control or individual HIV PIs (lopinavir, ritonavir, and atazanavir) at a clinically relevant concentration of 15 µM for 24 h. TNF-α and IL-6 protein levels in the medium were determined by ELISA as described previously [9]. As shown in Figure 1A and 1B, berberine significantly inhibited HIV PI-induced TNF-α and IL-6 expression at a concentration as low as 0.1 µg/ml. Berberine also reduced the basal expression levels of IL-6 and TNF-α at concentrations of 0.1 µg/ml and 1.0 µg/ml respectively.

### Effect of Berberine on HIV PI-Induced UPR Activation in Macrophages

**Figure 2:**
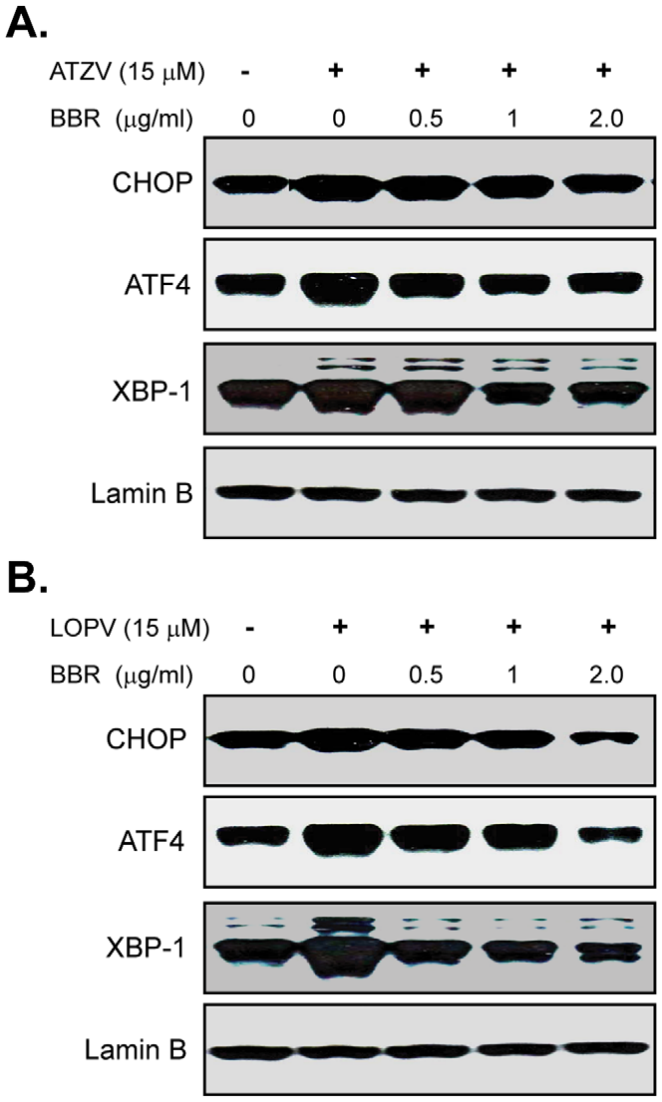
Effect of berberine on HIV PI-induced UPR activation in J774A.1 macrophages. Representative immunoblots from three independent experiments for CHOP, ATF-4, XBP-1 and lamin B from nuclear extracts of mouse J774A.1 cells pre-treated with various amounts of berberine (BBR, 0, 0.5, 1, 2.0 µg/ml) for 2 h, then treated with (**A**) atazanavir (ATZV, 15 µM); (**B**) lopinavir (LOPV, 15 µM) for 4 h. Lamin B was used as loading control.

It has become increasingly evident that activation of the UPR is closely linked to the development and progression of atherosclerotic lesions. Although the mechanisms by which ER stress inducers promote pro-atherogenic changes remain to be fully identified, it has been demonstrated that ER stress can induce the activation of inflammatory pathways [18]. We have previously shown that HIV PIs activate the UPR both in macrophages and hepatocytes [8], [19]. Our recent study also indicates that UPR activation contributes to the HIV PI-induced inflammatory response [10]. We further examined whether berberine has an inhibitory effect on HIV PI-induced UPR activation in macrophages. Mouse J774A.1 cells were pretreated with berberine for 2 h, and then treated with individual HIV PI for 4 h. As shown in Figure 2, berberine dose-dependently inhibited HIV PI-induced CHOP, XBP-1 and ATF-4 expression.

**Figure 3:**
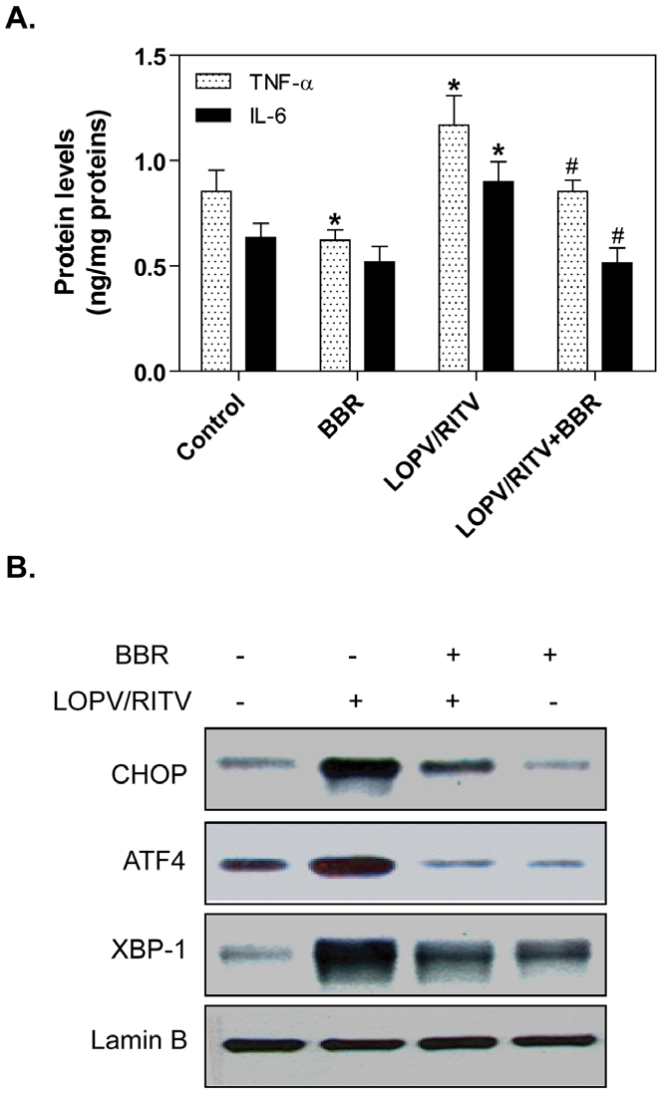
Effect of berberine on HIV PI-induced TNF-α and IL-6 expression and UPR activation in primary mouse macrophages. Primary mouse macrophages were pretreated with berberine (BBR, 2.0 µg/ml) for 2 h, then treated with lopinavir/ritonavir (LOPV/RITV = 4∶1, 15 µM) for 24 h. At the end of treatment, cell culture medium was collected. (A). The protein levels of TNF-α and IL-6 were determined by ELISA as described in “Methods”. Values are mean ± SD of 3 independent experiments. *p<0.05, statistical significance relative to vehicle control, DMSO. ^#^ *p*<0.05, statistical significance between LOPV/RITV and LOPV/RITV+BBR group. (B). Representative immunoblots for CHOP, ATF-4, XBP-1 and lamin B from nuclear extracts of primary mouse macrophages pre-treated with various amounts of berberine (BBR, 2.0 µg/ml) for 2 h, then treated with lopinavir/ritonavir (LOPV/RITV = 4∶1, 15 µM) for 24 h. Lamin B was used as loading control.

We further confirmed the effect of berberine on HIV PI-induced TNF-α and IL-6 expression and UPR activation using primary mouse macrophages. As shown in Fig. 3, treatment the mouse peritoneal macrophages with lopinavir/ritonavir (4∶1), the most commonly used HIV PIs in the clinic, not only significantly increased TNF-α and IL-6 expression, but also induced the UPR activation. Similar to the observation in cultured mouse macrophages, berberine prevented HIV PI-induced inflammatory cytokine expression and ER stress response in primary mouse macrophages.

### Effect of Berberine on HIV PI-Induced ERK Activation in Macrophages

**Figure 4:**
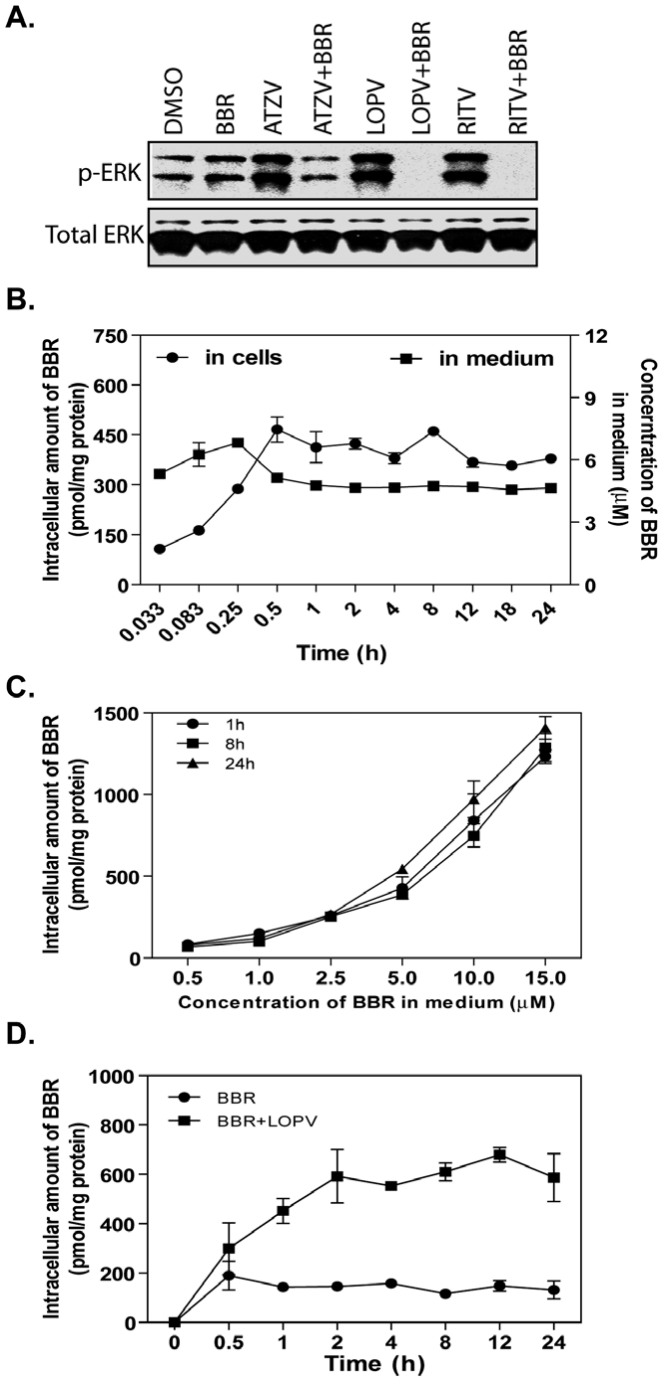
A. Effect of berberine on HIV PI-induced ERK activation in macrophages. Representative immunoblots from three independent experiments for phospho(p)-ERK and total (T)-ERK from the total cell lysates of mouse J774A.1 cells treated with berberine (BBR, 0.5 µg/ml) and individual HIV PIs (15 µM) for 4 h. ATZV: atazanavir; LOPV: lopinavir; RITV: ritonavir. **B: Time course of berberine uptake.** Mouse J774A.1 cells were treated with berberine (BBR, 5 µM) for various time periods (0 to 24 h), the concentration of BBR in culture medium and total intracellular amount were determined by HPLC as described in “Methods”. **C: Dose-dependence of berberine uptake in macrophages.** Cells were treated with various amounts of berberine (BBR, 0.5 to 15 µM) for 1, 8, or 24 h. The intracellular concentrations of BBR were determined as described above and expressed as pmol/mg protein. **D. Effect of lopinavir on berberine uptake in macrophages**. Mouse J774A.1 cells were treated with berberine (BBR, 5 µM) in the presence or absence of lopinavir (LOPV, 15 µM) for various time periods (0 to 24 h), the concentration of BBR in culture medium and total intracellular amount were determined by HPLC as described above.

The mitogen-activated protein kinase (MAPK) signaling pathways are involved in regulating the expression of many inflammatory genes [20]. It has been shown that accumulation of free cholesterol in macrophages induces ER stress and increases TNF-α and IL-6 expression by activating JNK, p38, and ERK signaling pathways [7]. Our recent studies found that ERK, but not p38 and JNK, is the key signaling pathway involved in HIV PI-induced inflammatory response, and HIV PI-induced UPR activation is responsible for ERK activation in macrophages [10]. It has been reported that berberine up-regulates the expression of hepatic low-density lipoprotein receptor by activating the ERK signaling pathway in HepG2 cells [21]. Berberine also inhibits 12-O-Tetradecanoylphorbol-13-acetate (TPA)-induced IL-6 expression in normal human keratinocytes by preventing ERK activation [22]. These studies suggest that berberine has a different effect on the ERK signaling pathway in different cell types. In order to delineate the potential signaling pathways underlying the inhibitory effect of berberine on HIV PI-induced TNF-α and IL-6 expression in macrophages, we further examined whether inhibition of HIV PI-induced UPR activation by berberine is correlated to its effect on HIV PI-induced ERK activation. As shown in Figure 4A, in the presence of berberine, HIV PI-induced ERK activation was completely inhibited. The p-ERK level in the cells treated with HIV PIs and berberine is even lower than control or berberine-treated cells. This suggests that HIV PIs may increase the uptake of berberine in macrophages. We further examined the effect of HIV PIs on berberine uptake in macrophages. As shown in Figure 4B, berberine was rapidly taken up by macrophages. The profile of the dose-dependent uptake of berberine at 1 h is similar to that of 24 h (Figure 4C). Although berberine had no effect on uptake of HIV PIs (data not shown), in the presence of lopinavir, the uptake of berberine was significantly increased (Figure 4D). These results suggest that berberine prevents HIV PI-induced inflammatory response by attenuating ER stress with subsequent inhibition of ERK activation in macrophages.

### Effect of Berberine on mRNA Stabilities of TNF-α and IL-6 in Lopinavir-Treated Macrophages

**Figure 5:**
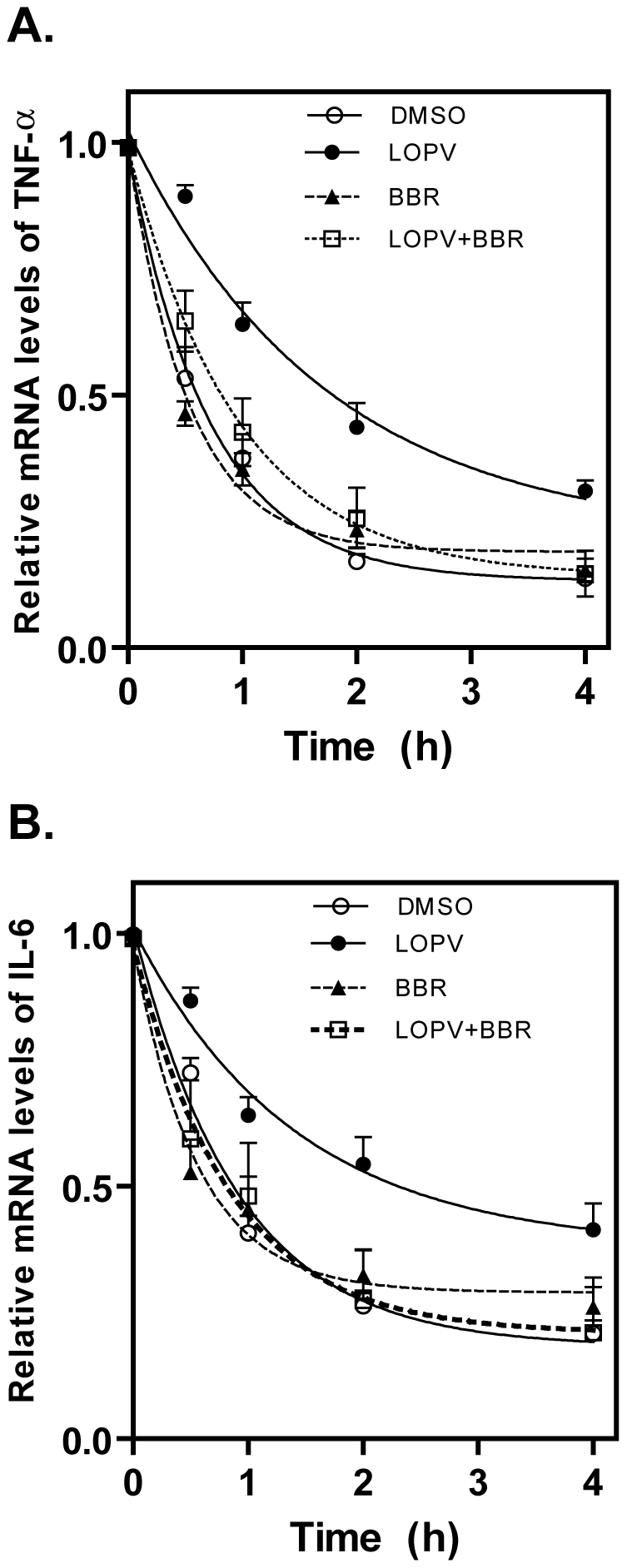
Effect of berberine on HIV PI-induced TNF-α and IL-6 mRNA stability in macrophages. Mouse J774A.1 cells were pretreated with berberine (BBR, 0.5 µg/ml) for 1 h, then treated with lopinavir (LOPV, 15 µM) or vehicle control (DMSO) for 4 h before addition of actinomycin D (5.0 µg/ml)(time 0). Total cellular RNA was extracted at 0, 0.5, 1, 2, and 4 h after actinomycin D addition. TNF-α (A) and IL-6 (B) mRNA levels were determined by real-time RT-PCR as described in “Methods”. Values are mean ± SD of three independent experiments.

Posttranscriptional regulation is a major control point for the expression of inflammatory cytokines. Our previous studies have shown that HIV PIs induce TNF-α and IL-6 expression by increasing mRNA stabilities [9]. In order to determine if berberine inhibits HIV PI-induced TNF-α and IL-6 expression by regulating the mRNA stabilities, we examined the effect of berberine on lopinavir-induced mRNA stabilization of TNF-α and IL-6. As shown in Figure 5, berberine completely blocked lopinavir-induced increase of TNF-α and IL-6 mRNA stability.

### Effect of Berberine on HIV PI-Induced Cytosolic Translocation of HuR and Its Binding to the mRNAs of TNF-α and IL-6 in Macrophages

**Figure 6:**
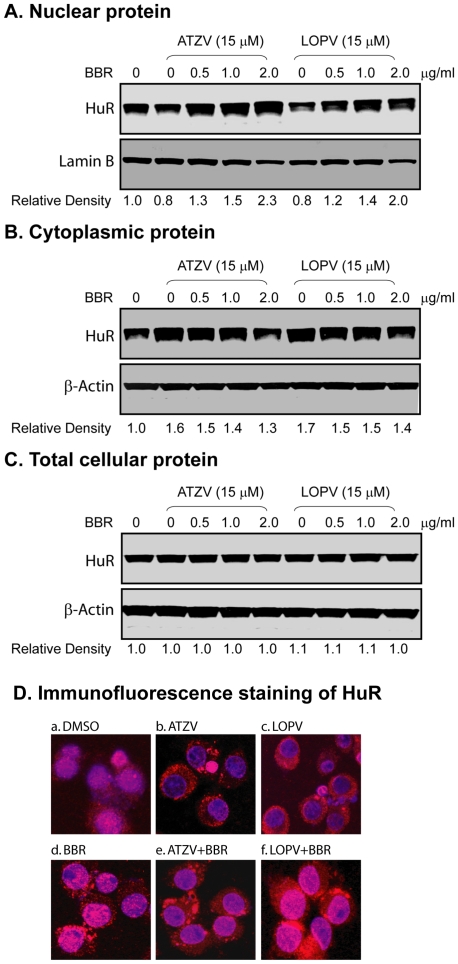
Effect of berberine on HIV PI-induced cytoplasmic translocation of RNA binding protein HuR in mouse macrophages. Mouse J774A.1 cells were pretreated with various amounts of berberine (BBR, 0, 0.5, 1, 2 µg/ml) for 2 h and then treated with atazanavir (ATZV, 15 µM) or lopinavir (LOPV, 15 µM) for 24 h. The nuclear proteins and cytoplasmic proteins were isolated as described in “Methods”. Three independent experiments were performed. Representative immunoblots against HuR from (A) nuclear proteins; (B) cytoplasmic proteins; (C) total cellular proteins. Lamin B and β-actin were used as loading controls for nuclear proteins and cytosolic/total cellular proteins. The density of the immunoreactive bands was analyzed using Image J software and normalized to lamin B or β-actin control. D. Immunofluorescent staining of cellular distribution of HuR. Cells were pretreated with berberine (BBR, 0.5 µg/ml) for 2 h and then treated with vehicle control DMSO, atazanavir (ATZV, 15 µM) or lopinavir (LOPV, 15 µM) for 24 h. Expression of HuR was detected with mouse monoclonal antibody against HuR and donkey anti-mouse IgG conjugated with Alex Fluor-594. Nuclei were stained with DAPI. HuR expression was visualized under a confocal fluorescence microscope with a 40× objective using a dual filter set for DAPI and rhodamine. Three experiments were performed that showed similar results.

We have demonstrated that HIV PI-induced stabilization of TNF-α and IL-6 mRNAs is mediated through regulating the translocation of RNA binding protein HuR from nucleus to cytosol and the binding of HuR to the 3′UTR of TNF-α and IL-6 mRNAs [9]. HIV PI-induced HuR cytosolic translocation is inhibited by both the MEK1 inhibitor PD98059 or over-expression of dominant negative MEK1 mutant [10]. Since berberine is a strong inhibitor of ERK activation in macrophages, we speculated that berberine should have a similar effect on HuR to the MEK1 inhibitor. We further examined the effect of berberine on HIV PI-induced cytosolic translocation of HuR. As shown in Figure 6A–C, berberine inhibited atazanavir and lopinavir-induced accumulation of cytosolic HuR, but had little effect on total cellular HuR levels. Immunofluorescence staining further verified our initial observation. As shown in Figure 6D, both atazanavir and lopinavir significantly increased HuR accumulation in cytosol, but in the presence of berberine, HuR was mainly translocated into the nucleus. As shown in online supplement Figure S1, berberine dose-dependently induced accumulation of HuR in the nucleus.

**Figure 7:**
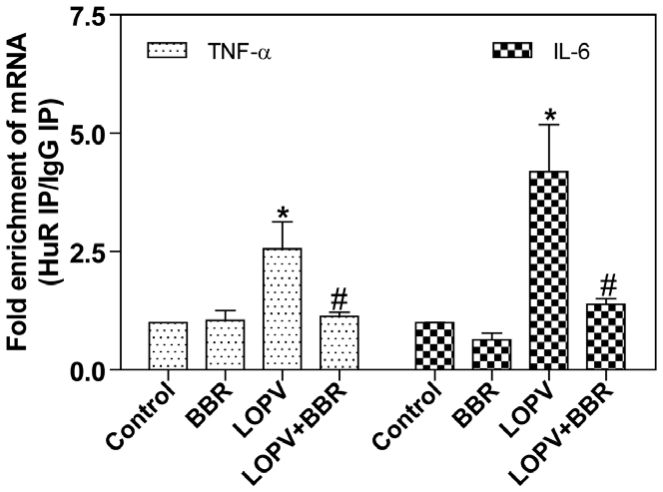
Effect of berberine on association of endogenous HuR with TNF-α and IL-6 in HIV PI-treated macrophages. Mouse J774A.1 cells were pretreated with berberine (BBR, 0.5 µg/ml) for 2 h and then treated with lopinavir (LOPV, 15 µM) for 24 h. Total cell lysates were subjected to immunoprecipitation (IP) using either a HuR Ab or an isotype-matched control IgG1. mRNA levels in IP materials were detected by real-time RT-PCR using gene-specific primers as described in “Methods” and expressed as fold enrichment of mRNA in HuR IP over control IgG1 IP. Values are mean ± SD of three independent experiments. Statistical significance relative to vehicle control, *p<0.05. Statistical significance of the same HIV PI treatment between control group and BBR group, ^#^ *p*<0.05.

We further examined if berberine also affected HIV PI-induced binding of HuR to TNF-α and IL-6 mRNAs by immunoprecipitating the endogenous HuR-mRNA complexes in macrophages as described previously [9]. As shown in Figure 7, lopinavir-induced increase of HuR binding to TNF-α and IL-6 mRNAs was completely blocked by berberine.

## Discussion

Drug-induced adverse effects not only cause significant health problems, but also become the most frequent single cause for termination of drug development and withdrawal of approved drugs from the market. Accumulating clinical evidence indicates that current anti-HIV therapy has changed the clinical profile of HIV infection from a sub-acute lethal disease to a chronic ambulatory disease [3], [23]. The development of other therapeutic interventions to counteract the HIV PI-induced cardiovascular complications is especially urgent.

In the history of drug discovery, natural products have provided an endless source of medicine and more than 60% of approved and pre-new drug application candidates are either natural products or related to them [24]. Traditional Chinese medicine (TCM) has been developed over thousands of years and has greatly contributed to human health [25]. It is also becoming more popular in the international society and mainly used in combinational therapies to reduce the side effects of conventional therapies. However, TCM mainly depends on clinical experience and its functional mechanism is poorly understood. The scientific evidence for the use of most of the TCM is largely lacking.

Berberine is the major component of Rhizoma coptidis (Huanglian), a herb that has been widely used to treat infectious diseases for more than 3,000 years [26]. During the last decade, berberine has been extensively studied and found to have various pharmacological activities including anti-inflammation, anti-oxidation, and anti-infection [27], [28]. It also has been shown that berberine has various beneficial effects on the cardiovascular system [29]. Furthermore, the most recent study has identified berberine as a novel cholesterol-lowering drug through a unique mechanism distinct from the current statin therapy [14]. In the present study, we provide new evidence showing that berberine prevents HIV PI-induced inflammatory response through modulating ER stress signaling pathways in macrophages.

ER stress response has emerged as an important cellular mechanism underlying various human diseases, especially for cardiovascular disease and metabolic syndrome [30], [31]. Our previous studies indicate that activation of the UPR plays a critical role in HIV PI-induced inflammatory response, dyslipidemia, and cardiovascular diseases [8], [9], [19]. The results reported here clearly show that berberine significantly inhibited HIV PI-induced TNF-α and IL-6 expression and UPR activation both in cultured and primary mouse macrophages (Figure 1–3). Recently, we further demonstrated for the first time that HIV PI-induced expression of inflammatory cytokines (TNF-α and IL-6) is coupled to the UPR and ERK signaling pathways [10]. In the current study, we found that berberine completely blocked HIV PI-induced ERK activation (Figure 4). Although berberine has been found to be able to regulate various signaling pathways including p-38 MAPK, PI-3 kinase, and AMPK under various physiological/pathological conditions, the effect of berberine on HIV PI-induced inflammatory response is mainly mediated through ER stress and ERK signaling pathways. In the CHOP knock out primary mouse macrophages, HIV PIs fail to activate the ERK signaling pathway [10].

The expression of inflammatory cytokines is tightly regulated through posttranscriptional mechanisms by modulating mRNA stability. The presence of AU-rich elements (AREs) in the 3′-untranslated region (3′UTR) is essential for the rapid decay of mRNAs of most short-lived inflammatory cytokines including TNF-α and IL-6 [32]. There is a growing body of evidence indicating that dysregulation of ARE-mediated mRNA decay contributes to chronic inflammation [32]. AREs mediate their regulatory function through association with multiple RNA binding proteins such as HuR, AUF1 and TTP [33], [34], [35], [36]. Our previous work demonstrated that HIV PIs increase TNF-α and IL-6 expression by regulating the intracellular translocation of HuR and its binding to 3′UTR of TNF-α and IL-6 [9]. Our most recent work further identified that HIV PI-induced ER stress response is responsible for ERK activation and subsequent increase of TNF-α and IL-6 mRNA stability and protein expression in macrophages [10]. In this study, we demonstrated that berberine exerts its anti-inflammatory effects through regulating the cytosolic translocation of RNA binding protein HuR and the binding of HuR to its target mRNAs. The pharmacokinetic studies further demonstrated that inhibition of HIV PI-induced ER stress and inflammatory response by berberine is not due to the inhibition of the uptake of HIV PIs in macrophages (Figure 4).

In summary, the current study identified a key cellular mechanism underlying the protective effect of berberine on HIV PI-induced inflammatory response in macrophages. Modulation of the ER stress response represents a potential therapeutic target for various inflammatory diseases and metabolic syndrome including HIV PI-associated atherosclerosis. The *in vivo* and clinical studies are needed to further determine the potential application of berberine as a complimentary agent for HIV infection.

## Materials and Methods

### Ethics Statement

Mice were housed and fed standard mouse chow and tap water ad libitum throughout the study following protocols approved by the IACUC at Virginia Commonwealth University.

### Materials

Antibodies against phospho-ERK1/2, ERK1/2, CHOP, XBP-1, ATF-4, HuR, β-actin, lamin B, horseradish peroxidase (HRP)-conjugated goat anti rabbit IgG, HRP-conjugated donkey anti goat IgG and HRP-conjugated goat anti mouse IgG were from Santa Cruz Biotechnology (Santa Cruz, CA). Berberine was purchased from Sigma (St. Louis, MO). Amprenavir, atazanavir, lopinavir, and ritonavir were obtained from NIH AIDS Research & Reference Reagent Program. Recombinant mouse TNF-α and IL6 were obtained from BioLegend (San Diego, CA). Anti-mouse TNF-α and IL-6 antibodies, biotinylated anti-mouse TNF-α and IL-6, and avidin-HRP were from eBioscience (San Diego, CA).

### Cell Culture and Treatment

Mouse J774A.1 macrophages were cultured as previously described [8]. HIV PIs (atazanavir, lopinavir, and ritonavir) and berberine were dissolved in dimethyl sulfoxide (DMSO) and directly added into the culture medium.

### Isolation of Mouse Peritoneal Macrophages

C57BL/6 wild type (male, 6–8 weeks old, Jackson Laboratories, Bar Harbor, ME) were injected intraperitoneally with 0.5 ml of phosphate-buffered saline (PBS) containing 40 µg concanavalin A. Mice were housed and fed standard mouse chow and tap water *ad libitum* throughout the study following protocols approved by the IACUC at Virginia Commonwealth University. The macrophages were harvested 72 h after injection by peritoneal lavage. The harvested cells were cultured in DMEM containing 10% fetal bovine serum (FBS) and 20% L-cell-conditioned medium [8]. The medium was replaced every 24 h until the macrophages were confluent.

### Quantification of Berberine Using High Performance Liquid Chromatography (HPLC) Assay

An Aglient 1200 Series HPLC system and a Beckman C18 reverse phase column (5 µm, 4.6 mm×25 cm) were used to quantify the berberine in cells and medium. The mobile phase was acetonitrile: water (30∶70, v/v) containing 0.08% formic acid and 0.15% ammonium acetate. The berberine was detected at a wavelength of 346 nm. A standard curve of berberine was constructed using weighted linear regression of peak area ratio values of the calibration standards.

Cells were treated with 5 µM of berberine for various time periods (0 to 14 h) or with different concentrations of berberine (0 to 15 µM) for 1, 8, and 24 h. Berberine in the cells and medium was extracted using methanol and subjected to HPLC analysis. The intracellular berberine was normalized with the total protein amount of viable cells.

### Western Blot Analysis

After treatment, total cell lysate, cytosolic proteins or nuclear proteins were prepared and used for Western blot analysis as described previously [8], [10]. The density of the immunoreactive bands was analyzed using Image J software (NIH) [8].

### Enzyme-Linked Immunosorbent Assay (ELISA) of TNF-α and IL-6

Cells were pre-treated with berberine for 2 h, and then treated with HIV PIs or vehicle control for 24 h. At the end of the treatment, culture media were collected and centrifuged at 14,000 x rpm for 1 min. TNF-α and IL-6 levels in the media were determined by ELISA as described previously [9]. The total protein concentrations of the viable cells were determined using Bio-Rad Protein Assay reagent. Total amounts of the TNF-α and IL-6 in media were normalized to the total protein amounts of the viable cell pellets and expressed as ng/mg proteins.

### RNA Isolation and Real-Time Quantitative RT-PCR

Total cellular RNA was isolated after treatment using the Promega SV Total RNA Isolation System. The first cDNA was synthesized using the High-Capacity cDNA Archive Kit. The mRNA levels of TNF-α and IL-6 were quantified with real-time PCR as described previously [9].

### Assessment of TNF-α and IL-6 mRNA Stability

Mouse J774A.1 macrophages were pretreated with berberine for 2 h, then treated with HIV PI (lopinavir, 15 µM) or vehicle control for 16 h before addition of actinomycin D (5.0 µg/ml) (time 0). Total cellular RNA was extracted 0.5, 1, 2, and 4 h after actinomycin D addition. TNF-α and IL-6 mRNA levels were determined by real-time RT-PCR as described in the previous section and results are expressed as the percentage of the mRNA amount at the time of actinomycin D addition.

### Immunofluorescent Staining

Mouse J774A.1 macrophages were treated with vehicle control (DMSO), lopinavir (15 µM) or atazanavir (15 µM) in the presence or absence of berberine for 24 h. Cells were fixed using 3.7% formaldehyde. The expression of HuR in the cytoplasm and nucleus was detected by immunofluorescent staining as described previously [9].

### Immunoprecipitation of Endogenous HuR-mRNA Complexes

To assess the effect of berberine on HIV PI-induced increase of association of endogenous HuR with endogenous TNF-α and IL-6 mRNAs, immunoprecipitation (IP) of endogenous HuR-mRNA complexes was performed as described previously [9].

### Statistical Methods

All data were expressed as mean ± SD. One-way ANNOVA was employed to analyze the differences between sets of data. Statistics were performed using GraphPad Pro (GraphPad, San Diego, CA). A value of *p*<0.05 was considered significant.

## Supporting Information

Figure S1 (0.05 MB PDF)
