# Supplementary material for: Berberine Inhibits HIV Protease Inhibitor-Induced Inflammatory Response by Modulating ER Stress Signaling Pathways in Murine Macrophages
Source: PLoS One. 2010 Feb 9;5(2):e9069. doi: 10.1371/journal.pone.0009069 (PMC2817721; doi:10.1371/journal.pone.0009069)
Supplement: Figure S1 — (0.05 MB PDF) [file pone.0009069.s001.pdf]

Figure S1

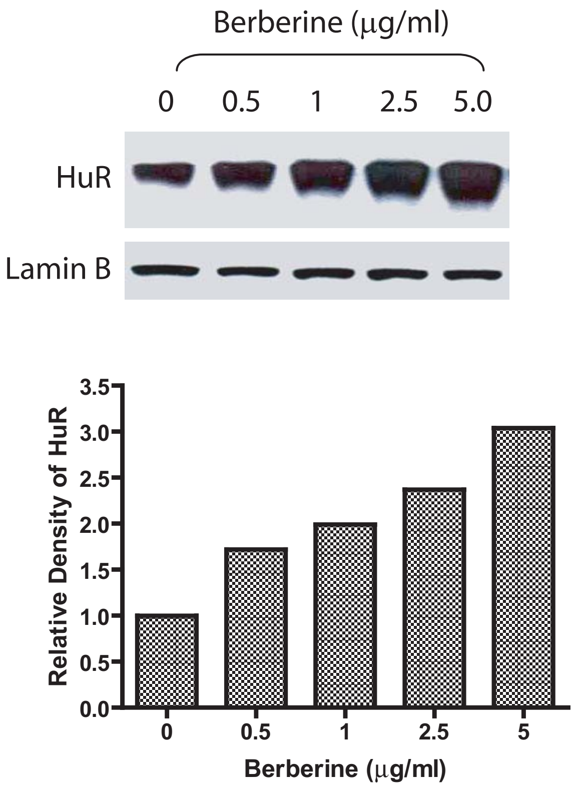

Effect of berberine on HuR accumulation in necleus in macrophages. Mouse J774A.1 cells were treated with different amount of berberine (0, 0.5, 1, 2.5, 5 μg/ml) for 24 h and the nuclear proteins were isolated. The protein levels of HuR in nucleus were determined by Western blot analysis. Lamin B was used as loading control for nuclear proteins.
